# Phosphate-Mediated Regulation of Intracellular Calcium Dynamics

**DOI:** 10.3390/cells15100901

**Published:** 2026-05-14

**Authors:** Huma Shahzad, Mohammed S. Razzaque

**Affiliations:** 1 Department of Medical Education, School of Medicine, University of Texas Rio Grande Valley (UTRGV), Edinburg, TX 78541, USA; 2 Center for Research and Innovation, Faculty of Medicine, Universiti Kuala Lumpur Royal College of Medicine Perak (UniKL RCMP), No. 3, Jalan Greentown, Ipoh 30450, Perak, Malaysia

**Keywords:** phosphate, calcium, FGF23, PTH, vitamin D

## Abstract

**Highlights:**

**What are the main findings?**

- Intracellular phosphate levels actively modulate calcium release, uptake, and distribution within the cell.
- Extracellular phosphate levels critically regulate calcium influx and overall cellular calcium handling.

**What are the implications of the main findings?**

- Tight crosstalk between phosphate and calcium implies that dysregulation of phosphate homeostasis may contribute to calcium-related pathologies, including arrhythmia, vascular calcification, muscle weakness, renal dysfunction, and bone disease.
- Therapeutic restoration of phosphate balance may improve the outcome of calcium-dependent pathologies.

**Abstract:**

Phosphate (Pi) and calcium (Ca^2+^) are essential mineral ions that play coordinated roles in maintaining normal cellular functions. While various steps of calcium signaling are well characterized, emerging evidence suggests the critical role of both intracellular and extra cellular phosphate in regulating intracellular Ca^2+^. In the cytoplasm, phosphate influences ATP production and organelle calcium buffering and influences the activity of calcium pumps, such as sarcoplasmic/endoplasmic reticulum Ca^2+^-ATPase (SERCA) and the plasma membrane Ca^2+^-ATPase (PMCA). Extracellular phosphate, taken up via sodium-dependent phosphate transporters, triggers signaling cascades that affect the processes of calcium influx, storage, and release. Additionally, high extracellular phosphate levels can disrupt calcium homeostasis through the systemic interactions of hormones such as fibroblast growth factor 23 (FGF23), vitamin D and parathyroid hormone (PTH), especially under pathological conditions such as chronic kidney disease (CKD). This article briefly summarizes the current understanding of the bidirectional influence of intra- and extracellular phosphate on calcium dynamics at the cellular level, with a focus on the underlying mechanisms.

## 1. Introduction

Calcium and phosphate are essential inorganic mineral ions that play diverse roles in cellular physiology. Calcium serves as a universal second messenger, and is involved in muscle contraction, neurotransmission, gene expression, and cell death pathways [1]. Optimal control of the intracellular Ca^2+^ concentration is critical, as slight fluctuations in cytosolic Ca^2+^ can disrupt the physiological balance to induce pathological cellular events, such as apoptosis, necrosis, or uncontrolled proliferation [2]. Phosphate, on the other hand, is indispensable for energy metabolism (as a key component of ATP), nucleic acid synthesis, cell signaling (via phosphorylation), and acid–base buffering [3,4]. Within cells, the availability of phosphate determines the energetic capacity of the cell and influences enzymatic and transporter functions, including those involved in intracellular Ca^2+^ handling.

Calcium and phosphate are systematically regulated by three key hormones: PTH, FGF23, and active vitamin D (1,25-dihydroxyvitamin D_3_). When extracellular calcium or phosphate levels are dysregulated, these hormones coordinate renal reabsorption, intestinal absorption, and bone remodeling to restore the mineral ion balance. For example, low serum calcium stimulates PTH release, which increases calcium reabsorption in the kidney and bone resorption, while it also promotes the activation of vitamin D to increase intestinal absorption. In contrast, high serum phosphate levels induce FGF23 production by bone cells, which decreases phosphate reabsorption in the kidney and suppresses vitamin D activation to reduce calcium and phosphate absorption [5,6]. These hormonal interactions ensure that calcium and phosphate balance remains tightly controlled in the circulation and is maintained within physiological limits to avoid pathological consequences, such as ectopic calcification or hypocalcemia-associated disorders.

The findings available point to phosphate-induced calcium regulation. In addition to classical endocrine regulation, intracellular phosphate directly modulates the machinery of calcium transport and buffering, whereas extracellular phosphate influences intracellular Ca^2+^ dynamics via transporter-mediated signaling and systemic hormonal feedback [4,7]. The importance of phosphate as an active modulator of intracellular Ca^2+^ homeostasis has significant implications for cellular physiology and disease conditions. The aim of this article is to provide a concise overview of the regulatory role of phosphate in maintaining intracellular Ca^2+^ homeostasis. By highlighting the delicate interplay between phosphate and calcium signaling, this article seeks to establish a conceptual framework that can guide further investigation. Finally, a deeper understanding of this relationship may lay the foundation for the development of targeted therapeutic strategies aimed at modulating calcium–phosphate interactions in both physiological and pathophysiological situations.

## 2. Effects of Intracellular Phosphate on Cellular Calcium Dynamics

**Figure 1:**
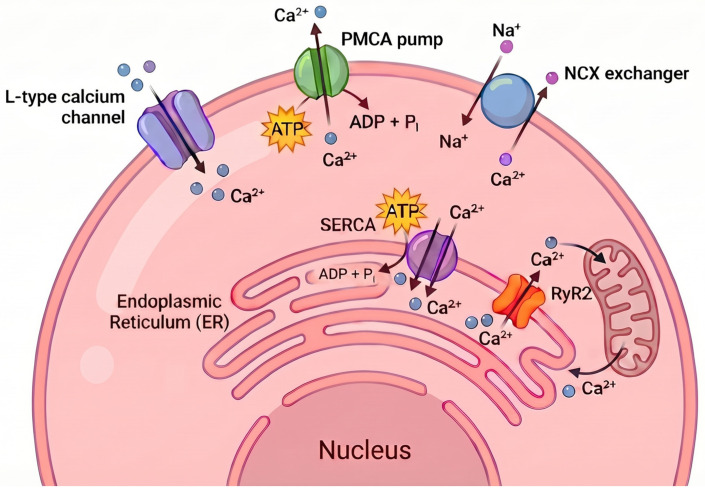
Simplified diagram of intracellular Ca^2+^ regulation and the use of phosphate in the form of ATP in such regulation. NCX: Na^+^/Ca^2+^ Exchanger; PMCA: Plasma Membrane Calcium ATPase; SERCA: Sarcoplasmic Reticulum Ca^2+^-ATPase; RyR2: Ryanodine Receptor 2 [10,11,12].

Intracellular phosphate is a key component of cellular metabolism and plays an important role in regulating intracellular Ca^2+^ homeostasis through its contributions to energy production and organelle function. One of the most fundamental roles of phosphate is as a substrate in ATP synthesis, both in glycolysis and in oxidative phosphorylation. Adequate ATP levels are essential for the function of key calcium-transporting ATPases, such as SERCA and PMCA (Figure 1) [8,9]. These pumps maintain low cytosolic Ca^2+^ concentrations by transporting calcium back into intracellular stores (such as the endoplasmic reticulum) or by transporting it out of the cell. A reduction in intracellular phosphate levels, and consequently ATP production, impairs the efficiency of these calcium pumps, leading to cytosolic Ca^2+^ accumulation and disruption of calcium signaling.

**Figure 2:**
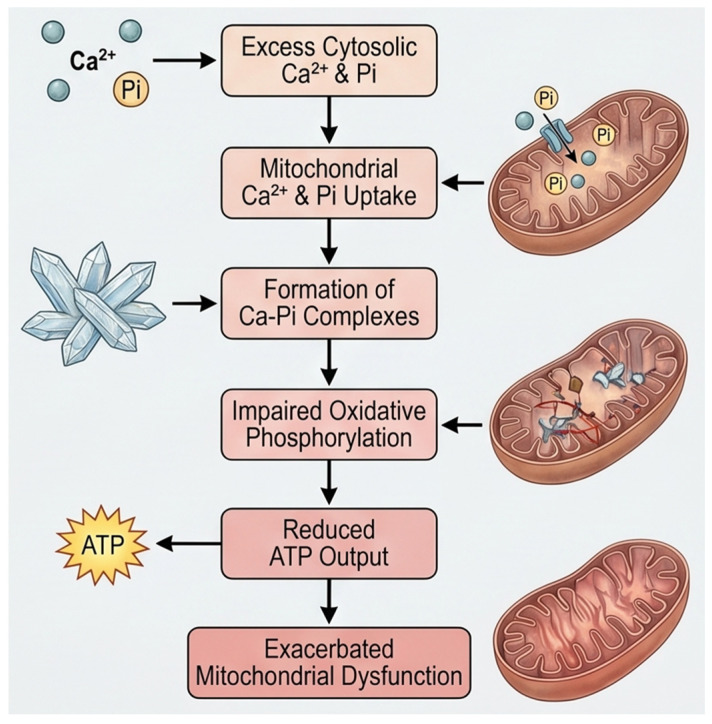
Simplified diagram illustrating how excessive accumulation of calcium–phosphate complexes in mitochondria disrupts their function.

In addition to its role in energy metabolism, intracellular phosphate also profoundly influences organelle-specific calcium handling, particularly within the mitochondria and the endoplasmic reticulum. In mitochondria, phosphate influences calcium uptake to form calcium–phosphate complexes that serve as a buffering system within the mitochondrial matrix [13,14]. Mitochondrial calcium overload occurs when calcium uptake exceeds the buffering capacity of organelles, typically because of sustained cytosolic Ca^2+^ elevation [15]. As the load increases, mitochondria lose function, generate excess ROS, and may undergo mitochondrial permeability transition pore (mPTP) opening, membrane injury, and eventual structural failure [14]. Intracellular phosphate facilitates this process by binding excess matrix calcium to form calcium–phosphate complexes or granules. This buffering is initially protective, but excessive granule formation can crowd the matrix, distort cristae, impair metabolite diffusion, and suppress oxidative phosphorylation, particularly complex I-linked respiration [16]. With further overload, these calcium–phosphate deposits contribute to inner membrane fragmentation, cristae remodeling, ATP depletion, and progression toward the mPTP. Clinically, mitochondrial Ca^2+^ overload is implicated in conditions such as heart failure, ischemia–reperfusion injury, and neurodegenerative diseases including Huntington’s disease and Parkinson’s disease [17,18]. Overall, mitochondrial Ca^2+^ overload, which is amplified by intracellular phosphate, drives mitochondrial structural damage and bioenergetic collapse (Figure 2).

Within the endoplasmic reticulum, phosphate availability influences calcium storage capacity by controlling the activity of SERCA pumps, which depend on ATP for their function [19]. Intracellular phosphate can enter the sarcoplasmic reticulum and interact with Ca^2+^ to form calcium–phosphate precipitates. This reaction effectively reduces the availability of free calcium within the sarcoplasmic reticulum, as the formation of calcium–phosphate complexes limit the release of Ca^2+^ during action potentials. Consequently, this precipitation process can diminish the action potential-mediated Ca^2+^ release, which is critical for muscle contraction. Specifically, as phosphate levels rise, it can lead to a decrease in the efficiency of the calcium release channels in the calcium–phosphate, which are modulated by phosphorylation and dephosphorylation processes [20].

Low intracellular phosphate levels result in ATP depletion, reduce SERCA activity and thereby deplete endoplasmic reticulum calcium stores. This can induce endoplasmic reticulum stress, leading to unfolded protein response (UPR) activation and calcium leakage into the cytosol via leaky channels such as inositol 1,4,5-trisphosphate receptors (IP3Rs) or ryanodine receptors. Chronic endoplasmic reticulum stress and calcium leakage compromise cellular viability and can trigger apoptotic pathways, particularly in metabolically active cells such as pancreatic β-cells, cardiomyocytes, and neurons [21]. In contrast, elevated intracellular phosphate levels, often observed in pathological states such as ischemia-reperfusion injury or metabolic acidosis, can lead to calcium overload, especially in mitochondria. These conditions disrupt the mitochondrial membrane potential, hinder oxidative phosphorylation, and initiate proapoptotic signaling. In this way, both low and high intracellular phosphate levels disrupt the delicate balance of intracellular Ca^2+^ homeostasis, with low phosphate levels leading to calcium depletion and endoplasmic reticulum dysfunction and high phosphate levels contributing to calcium overload, oxidative stress, and mitochondrial damage. These organelle-specific effects of intracellular phosphate highlight its key role as a regulator of intracellular Ca^2+^ homeostasis. As both calcium and phosphate share strong physical and metabolic interdependence, maintaining optimal intracellular phosphate levels is essential not only for energy supply but also for calcium buffering, storage, and signaling networks across cell types.

Mechanistically, phosphate influences Ca^2+^ homeostasis through direct effects, such as calcium–phosphate complex formation and modulation of Ca^2+^ transport systems, as well as mitochondrial pathways. Intracellular phosphate regulates Ca^2+^ homeostasis through direct binding and mitochondrial mechanisms. Phosphate forms calcium–phosphate complexes that transiently buffer cytosolic Ca^2+^. In mitochondria, phosphate uptake via the mitochondrial phosphate carrier (PiC) supports Ca^2+^ sequestration through the mitochondrial calcium uniporter (MCU). The resulting calcium–phosphate complex formation reduces free matrix Ca^2+^, maintaining the electrochemical gradient for continued Ca^2+^ uptake and enhancing mitochondrial buffering capacity. In the endoplasmic reticulum, phosphate regulates Ca^2+^ handling by influencing the ATP availability required for the SERCA pump, thereby affecting Ca^2+^ reuptake and modulating release via the IP3Rs and ryanodine receptors. In the plasma membrane, phosphate levels are controlled by NaPi transporters to influence Ca^2+^ dynamics. Phosphate-dependent changes in cellular energy status alter Na^+^ gradients, thereby regulating Ca^2+^ extrusion through the NCX. Phosphate modulates Ca^2+^ homeostasis by activating intracellular signaling pathways, including the PI3K/Akt signaling pathway and MAPK/ERK pathway, which regulate the phosphorylation and activity of Ca^2+^ channels and transporters. Additionally, phosphate influences ion channel function by altering ATP-dependent kinase activity, affecting channels such as L-type calcium channels and SOLC components, including STIM1 and ORAI1. Together, these mechanisms underscore the essential role of intracellular phosphate in fine-tuning Ca^2+^ homeostasis at the cellular level.

## 3. Effects of Extracellular Phosphate on Cellular Calcium Dynamics

Extracellular phosphate plays a key role in modulating intracellular Ca^2+^ homeostasis through both direct cellular events and the modulation of systemic pathways. Cells take up phosphate mainly via sodium-dependent phosphate transporters (NaPi2a, NaPi2b, NaPi2c, PiT-1 and PiT-2), which are widely expressed in various tissues. Upon phosphate uptake, these transporters not only supply phosphate for cellular metabolism but also activate intracellular signaling cascades such as the mitogen-activated protein kinase/extracellular signal-regulated kinase (MAPK/ERK) and phosphoinositide 3-kinase/protein kinase B (PI3K/Akt) pathways [22]. These signaling events can alter the activity of various calcium channels, including voltage-gated calcium channels, transient receptor potential (TRP) channels, and store-operated calcium entry (SOCE) channels, resulting in changes in calcium influx across the plasma membrane. In addition, high extracellular phosphate levels can modulate the sensitivity of calcium-sensing receptors (CaSRs) on cell surfaces, influencing intracellular Ca^2+^ signaling and downstream cellular responses [23]. Through these combined effects, extracellular phosphate exerts delicate control over intracellular Ca^2+^ levels to maintain the physiological function of cells and prevent pathological mineral deposition.

Although extracellular phosphate can directly influence cellular calcium dynamics through transporter-mediated signaling, much of its effect on intracellular Ca^2+^ homeostasis is also influenced by systemic hormonal responses. These responses are tightly regulated by endocrine networks involving FGF23, PTH, and vitamin D, which collectively ensure that calcium and phosphate levels remain balanced in both the intracellular and extracellular compartments. One of the first systemic responses to elevated extracellular phosphate is an increase in the expression of FGF23, a phosphate-regulating hormone secreted primarily by bone cells [24]. FGF23 acts on the kidneys to promote phosphate excretion and to suppress the activity of renal 1α-hydroxylase, the enzyme responsible for converting inactive vitamin D into its active form, calcitriol (1,25-dihydroxyvitamin D_3_) [5]. A reduction in calcitriol levels leads to diminished intestinal absorption of calcium, thereby lowering serum calcium concentrations. In response to this hypocalcemia, the parathyroid glands secrete more PTH, which acts to restore calcium levels by stimulating bone resorption, enhancing renal calcium reabsorption, and promoting residual vitamin D activation. This hormonal loop allows the body to protect calcium availability at the cost of altering bone integrity and shifting calcium balance across tissues.

These endocrine responses have important downstream effects on intracellular Ca^2+^ regulation. For example, increased PTH levels can influence intracellular Ca^2+^ signaling in target tissues such as bone, kidney, and vascular smooth muscle by activating PTH receptors linked to the cAMP and phospholipase C (PLC) pathways, leading to increased cytosolic Ca^2+^. Simultaneously, reduced calcitriol limits calcium uptake by enterocytes and other epithelial cells, altering their intracellular Ca^2+^ pool and potentially disrupting calcium-dependent signaling. Over time, this vicious cycle, driven by persistently high phosphate levels, can lead to chronically elevated PTH levels (secondary hyperparathyroidism) and maladaptive intracellular Ca^2+^ regulation, especially in cells sensitive to hormonal activation, such as osteoblasts, vascular smooth muscle cells (VSMCs), and cardiomyocytes. These dynamics form a systemic feedback loop in which extracellular phosphate acts as a primary stimulus that alters the activity of hormonal regulators, in turn altering tissue-level calcium handling and intracellular Ca^2+^ homeostasis. Importantly, while this loop is initially protective, chronic dysregulation, as seen in conditions such as CKD, can shift this adaptive mechanism toward pathology. In such states, sustained elevations in FGF23 and PTH, coupled with suppressed vitamin D, lead to disordered mineral metabolism, vascular calcification, and cardiac remodeling, all of which are driven partly by altered intracellular Ca^2+^ dynamics linked to systemic phosphate overload.

From the available information, it is apparent that extracellular phosphate influences intracellular Ca^2+^ not only through transporter activity or signaling cascades but also through complex hormonal feedback systems [25,26]. These feedback loops are key to the mineral ion balance of the body, and when disrupted, they form a pathophysiological link between systemic phosphate imbalance and calcium-driven cellular injury.

## 4. Physiological and Pathophysiological Implications

Under homeostatic conditions, phosphate-dependent calcium regulation supports essential cellular and tissue functions, such as muscle contraction, signal transduction, and the regulated secretion of neurotransmitters. In excitable cells, such as cardiomyocytes, neurons, and skeletal muscle fibers, adequate control of the intracellular Ca^2+^ level is required for rhythmic contraction and neurotransmission. Secretory pathways, such as hormone or neurotransmitter release, rely on rapid, transient increases in intracellular Ca^2+^, which are modulated by phosphate-dependent energy production and calcium reuptake mechanisms. Together, phosphate and calcium dynamically cooperate to regulate cellular excitability, metabolism, and communication.

**Figure 3:**
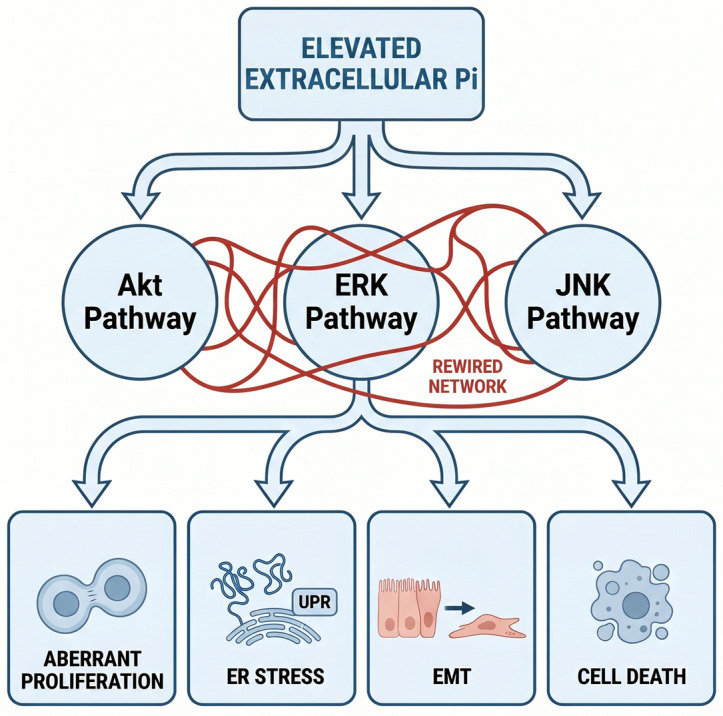
Simplified diagram illustrating the cytotoxic effects of elevated extracellular Pi through the reprogramming of interconnected signaling networks [30]. ER stress: Endoplasmic Reticulum stress; EMT: Epithelial-Mesenchymal Transition.

However, in pathological contexts, particularly where phosphate levels are elevated or dysregulated, this finely tuned balance is disrupted, leading to cytotoxicity and organ dysfunction. In CKD, reduced renal excretion of phosphate leads to persistent hyperphosphatemia, which initiates a cascade of hormonal and cellular responses detrimental to calcium homeostasis. Elevated phosphate levels stimulate FGF23 secretion and suppress vitamin D activation, reducing intestinal calcium absorption and promoting secondary hyperparathyroidism. These systemic responses increase bone resorption and increase circulating calcium and phosphate levels, indicating that ectopic calcium–phosphate crystal deposition occurs in soft tissues and blood vessels [27]. High extracellular phosphate levels drive vascular calcification by promoting osteogenic reprogramming in VSMCs, where sodium-dependent phosphate cotransporters (Pit-1/Pit-2) facilitate uptake, elevating intracellular phosphate levels and activating signaling cascades such as ERK, mTOR, TLR4/NF-κB, and oxidative stress pathways that downregulate the expression of contractile markers [e.g., smooth muscle 22 alpha (SM22α), and alpha-smooth muscle actin (α-SMA)] while upregulating the expression of osteogenic transcription factors such as Runx2, Cbfa1, osterix, and bone morphogenetic protein 2 (BMP-2). This phenotypic switch induces the expression of bone-related proteins (ALP, osteopontin, osteocalcin), facilitating hydroxyapatite deposition in the extracellular matrix; the whole process is further compounded by intracellular Ca^2+^ influx via SOCE, which is primarily meditated by stromal interaction molecule 1 (STIM1)/calcium release-activated calcium modulator 1 (ORAI1) [28]. Consequently, vascular calcification increases arterial stiffness and heightens cardiovascular mortality and morbidity, particularly in CKD patients with hyperphosphatemia [29]. At the cellular level, high levels of phosphate disrupt mitochondrial calcium buffering and endoplasmic reticulum calcium handling, contributing to calcium overload, oxidative stress, and apoptotic cell death, particularly in cardiovascular tissues. Studies performed on HEK293 and HeLa cell lines have demonstrated that elevated extracellular phosphate exposure can remodel complex networks of intracellular signaling, particularly the Akt, ERK, and c-Jun N-terminal kinase (JNK) pathways, thereby promoting a spectrum of cytotoxic responses, including aberrant cell proliferation, endoplasmic reticulum stress, epithelial–mesenchymal transition (EMT), and cell death (including apoptosis and necrosis) (Figure 3) [30].

In addition to CKD, dysregulated phosphate–calcium interactions are also implicated in neurodegeneration and heart diseases. Neurons are especially vulnerable to imbalances in calcium homeostasis because of their reliance on tightly regulated calcium signaling for synaptic function, plasticity, and survival. Elevated intracellular phosphate, or disturbed mitochondrial calcium buffering due to high phosphate load, can promote excitotoxicity, mitochondrial dysfunction, and neuronal death, processes linked to neurodegenerative disorders [31,32,33]. Calcium–phosphate-induced neuronal injury primarily occurs through the acute deposition of calcium–phosphate nanoparticles, particularly under ischemic conditions, where elevated intracellular Ca^2+^ and phosphate levels, often triggered by plasma membrane degradation, lead to the release of neuron-specific annexins, which form Annexin/calcium–phosphate complexes that bind β-actin filaments, disrupting cytoskeletal integrity, signal transduction, synaptic plasticity, and axonal dynamics prior to infarction. This deposition exacerbates Ca^2+^ overload, activating damaging enzymes (e.g., phospholipases, proteases, calpains, and nucleases), mitochondrial mPTP opening via phosphate–polyphosphate interactions, ROS surge, ATP depletion, inflammation, and DNA damage, culminating in necrosis or apoptosis; however, low-density free calcium–phosphate may initially buffer cytosolic Ca^2+^ cytotoxicity, and high-density structural deposition shifts to neurotoxic effects [34,35]. Chronic calcium–phosphate accumulation contributes to neurodegeneration, as seen in primary familial brain calcification (PFBC) and phosphate toxicity models, manifesting as cognitive decline (memory loss, concentration deficits), psychiatric disturbances (psychosis, personality changes), seizures, movement disorders (parkinsonism, dystonia), and dementia-like syndromes via sustained neuroinflammation, the potential of amyloid-β/tau pathology, and impaired neuronal regeneration. In Parkinson’s disease, phosphate impairs mitochondrial complex I, amplifies mPTP opening, and promotes α-synuclein aggregation, whereas stroke-related calcium–phosphate is correlated with persistent vascular dysfunction and heightened stroke recurrence risk [34,36].

Similarly, in the heart, chronic exposure to high phosphate levels can lead to cardiac hypertrophy, fibrosis, and arrhythmias, likely due to altered calcium cycling in cardiomyocytes and maladaptive hormonal signaling (e.g., excess PTH and FGF23) [37,38]. Chronic exposure to high phosphate levels in the heart promotes calcium–phosphate deposition and disrupts calcium cycling in cardiomyocytes, leading to cardiac hypertrophy, fibrosis, and arrhythmias through interconnected molecular pathways. Elevated extracellular phosphate drives the formation of calcium pyrophosphate particles (CPPs) and nanoparticles, which enter cardiomyocytes via endocytosis or paracellular leakage, and dissolve in acidic lysosomes to release Ca^2+^ and phosphate; this triggers lysosomal proton pump (v-ATPase)-dependent cytosolic Ca^2+^ overload, activating calpains, phospholipases, and mPTP opening, resulting in ROS production, ATP depletion, and cardiomyocyte apoptosis or necrosis. Concurrently, hyperphosphatemia upregulates PTH/PTH1R pathways (enhancing Ca^2+^ influx via altered SERCA/Na^+^/Ca^2+^ exchanger (NCX)/RyR2 handling and phospholamban hyperphosphorylation), promoting maladaptive hypertrophy via calcineurin-NFAT and MAPK/ERK activation, extracellular matrix remodeling (TGF-β, collagen upregulation), and arrhythmogenic afterdepolarization from sarcoplasmic reticulum Ca^2+^ leakage [37,39,40,41]. Persistent calcium–phosphate-induced injury culminates in left ventricular hypertrophy (LVH), myocardial and valvular fibrosis, diastolic dysfunction, increased aortic stiffness, and heightened risk of atrial fibrillation, heart failure, and sudden cardiac death, particularly in CKD patients where CPPs correlate with cardiovascular events independent of traditional risk factors. A decrease in therapeutic phosphate (e.g., binders, diet) may delay progression by reducing CPP formation and hormone dysregulation [39,40].

High extracellular phosphate also induces skeletal muscle injury through multiple interconnected molecular mechanisms, primarily by impairing ATP generation via mitochondrial dysfunction and sodium–phosphate co-transporter-mediated uptake, which elevates intracellular phosphate levels, reduces oxidative phosphorylation, and accelerates phosphocreatine depletion and increases ADP accumulation. Concurrently, it promotes calcium–phosphate precipitation, which disrupts myofibrillar integrity, excitation–contraction coupling, and contractility, while enhancing the overproduction of ROS, which activates Nrf2 signaling, through both Keap1 dissociation and p62-mediated pathways, suppressing myogenin activity, promoting proteasomal atrophy, and inhibiting myogenic differentiation. In muscle pathologies, these effects coordinate with PLC/IP3/Ca^2+^ pathway hyperactivation, amplifying sarcoplasmic reticulum Ca^2+^ release, cytosolic overload, calpain activation, and myocyte degeneration, with reduced klotho levels exacerbating energy deficits during exercise and overall weakness [42,43,44,45]. Thus, phosphate-induced calcium dysregulation emerges as a convergent mechanism underlying pathology across multiple organ systems. In a similar line of study, hyperphosphatemia was shown to trigger systemic inflammation via NFκB activation in hepatocytes leading to elevated proinflammatory cytokines (e.g., IL-6) and hepcidin, exacerbating functional iron deficiency (anemia) and skeletal muscle wasting in CKD mouse models and high-phosphate-diet-fed mice [46].

These findings have generated interest in therapeutic strategies aimed at restoring calcium–phosphate balance. In CKD, phosphate binders (e.g., sevelamer and lanthanum carbonate) are typically used to limit intestinal phosphate absorption and reduce serum phosphate levels. Dietary phosphate restriction, particularly in terms of reducing the intake of highly absorbable inorganic phosphate additives found in processed foods, has been shown to be beneficial for lowering circulating phosphate levels and improving cardiovascular outcomes. Emerging strategies include targeting phosphate transporters and inhibiting downstream signaling pathways [e.g., ERK1/2, and nuclear factor of activated T cells (NFAT)] that mediate the cellular effects of phosphate overload [47]. There is also growing interest in modulating FGF23 signaling, although balancing its beneficial phosphate-lowering effects against potential cardiotoxicity remains a challenge [48]. Ultimately, therapeutic efforts that correct excess phosphate or disrupt maladaptive calcium–phosphate signaling may offer new opportunities to prevent or slow the progression of cardiovascular and neurodegenerative diseases [49,50].

## 5. Conclusions and Future Directions

The regulation of intracellular Ca^2+^ by phosphate represents a complex and finely balanced system involving both intracellular and extracellular phosphate pools. Intracellular phosphate is essential for maintaining calcium homeostasis through its roles in ATP synthesis, mitochondrial buffering, and phosphorylation-dependent signaling, all of which support the activity of calcium transporters and channels (Table 1). Extracellular phosphate, in turn, affects intracellular Ca^2+^ dynamics both directly via phosphate transporters that activate intracellular signaling pathways and through systemic hormonal regulators such as FGF23, PTH, and vitamin D. Together, these mechanisms ensure the tight control of cellular calcium levels across a variety of tissues.

Despite growing insights into this interplay, major gaps remain. The organ- and cell type-specific consequences of altered calcium–phosphate regulation are poorly understood, particularly in nonclassical tissues such as the brain, immune system, or endocrine organs. Furthermore, the crosstalk between phosphate and calcium transporters and how they integrate with other signaling networks (e.g., oxidative stress, mTOR, or inflammatory pathways) are largely unexplored. Additionally, the relative contributions of acute and chronic phosphate exposure to cellular calcium dysregulation and pathology are still unclear. A key physiological gap is that intracellular phosphate sensing and compartment-specific buffering remain poorly defined, including how phosphate signals are translated into adaptive regulation of transporters and mitochondrial functions [55]. A parallel pathological gap is that phosphate can accumulate within intracellular compartments, such as mitochondria, even when serum levels are only modestly elevated or are intermittently abnormal, indicating that local phosphate handling may be more consequential than plasma concentrations alone. This may explain why tissues such as the kidney, heart, muscle, and neurons sustain injury despite relatively mild systemic disturbances, and suggests that calcification and dysfunction reflect cell-intrinsic phosphate dysregulation rather than solely a whole-body imbalance [56]. Moreover, estrogen regulates NaPi-2a expression in renal tubules, thereby influencing urinary phosphate excretion. However, despite evidence detailing its effects on extracellular (serum) phosphate levels, a significant gap remains in understanding how estrogen regulates intracellular phosphate concentrations [57,58].

Eventually, there is strong potential for therapeutically targeting phosphate-calcium interactions in diseases characterized by mineral imbalance, most notably CKD and cardiovascular diseases, where phosphate retention and calcium overload drive vascular calcification and cardiac dysfunction. Modulating phosphate transporter activity, enhancing phosphate clearance, or disrupting maladaptive hormonal feedback loops could offer new strategies to restore mineral balance and prevent tissue injury. Future research that clarifies these mechanisms across different organs and disease states will be essential to translate these insights into clinically useful interventions.

## Figures and Tables

**Table 1:** Effects of intracellular phosphate on cellular calcium dynamics [11,23,51,52,53,54]. ↑ = increase; ↓ = decrease.

Category	Examples	Main Locations	Role on Calcium	Effects of Intra-Cellular Phosphate
**NMDAR**	✓ GluN1/GluN2A ✓ GluN1/GluN2B ✓ GluN1/GluN2D	▪ Postsynaptic neurons (brain, spinal cord) ▪ Some peripheral tissues	Permeable channel—lets Ca^2+^ in when activated	High intracellular phosphate (as ATP/high-energy form) required for channel function; depletion causes rundown/loss of currents Phosphorylation by kinases modulates activity
**Calcium channels**	✓ Caᵥ1.2 (L-type) ✓ Caᵥ2.1 (P/Q-type) ✓ Orai1 (store-operated)	▪ Muscles ▪ Neurons ▪ Immune cells	Voltage/store-gated channels—open to allow Ca^2+^ entry	Can support channel modulation via phosphorylation High levels aid Ca^2+^ buffering
**Calcium receptors**	✓ CaSR (calcium-sensing receptor)	▪ Parathyroid ▪ Kidney ▪ Bone	Extracellular sensor—detects Ca^2+^ levels for homeostasis	↑ Phosphate → ↓ CaSR sensitivity → ↑ PTH secretion ↓ Phosphate → ↑ CaSR responsiveness → stronger calcium signaling
**Calcium pumps**	✓ PMCA ✓ SERCA ✓ SPCA	▪ Plasma membrane ▪ ER/SR ▪ Golgi	ATP-driven exporters—pump Ca^2+^ out/into stores	Rely on intracellular phosphate (ATP) for energy High phosphate may enhance activity via metabolism

## Data Availability

No new data were created or analyzed in this study. Data sharing is not applicable to this article.
